# Influence of Edaphic, Climatic, and Agronomic Factors on the Composition and Abundance of Nitrifying Microorganisms in the Rhizosphere of Commercial Olive Crops

**DOI:** 10.1371/journal.pone.0125787

**Published:** 2015-05-07

**Authors:** Joan Caliz, Miguel Montes-Borrego, Xavier Triadó-Margarit, Madis Metsis, Blanca B. Landa, Emilio O. Casamayor

**Affiliations:** 1 Biogeodynamics & Biodiversity Group, Centro de Estudios Avanzados de Blanes, CEAB-CSIC, Blanes, Girona; 2 Institute for Sustainable Agriculture, Spanish National Research Council (CSIC), Campus de Excelencia Internacional Agroalimentario (ceiA3), Córdoba, Spain; 3 Tallinn University, Institute of Mathematics and Natural Sciences, Narva mnt 25, Tallinn 10120, Estonia; University of Freiburg, GERMANY

## Abstract

The microbial ecology of the nitrogen cycle in agricultural soils is an issue of major interest. We hypothesized a major effect by farm management systems (mineral versus organic fertilizers) and a minor influence of soil texture and plant variety on the composition and abundance of microbial nitrifiers. We explored changes in composition (16S rRNA gene) of ammonia-oxidizing archaea (AOA), bacteria (AOB), and nitrite-oxidizing bacteria (NOB), and in abundance of AOA and AOB (qPCR of amoA genes) in the rhizosphere of 96 olive orchards differing in climatic conditions, agricultural practices, soil properties, and olive variety. Majority of archaea were 1.1b thaumarchaeota (soil crenarchaeotic group, SCG) closely related to the AOA genus *Nitrososphaera*. Most AOB (97%) were identical to *Nitrosospira tenuis* and most NOB (76%) were closely related to *Nitrospira* sp. Common factors shaping nitrifiers assemblage composition were pH, soil texture, and olive variety. AOB abundance was positively correlated with altitude, pH, and clay content, whereas AOA abundances showed significant relationships with organic nitrogen content and exchangeable K. The abundances of AOA differed significantly among soil textures and olive varieties, and those of AOB among soil management systems and olive varieties. Overall, we observed minor effects by orchard management system, soil cover crop practices, plantation age, or soil organic matter content, and major influence of soil texture, pH, and olive tree variety.

## Introduction

Reactive nitrogen (Nr), the forms of N that have a reduced oxidation state relative to N_2_ (mostly NO_3_^-^ and NH_4_^+^), is an essential nutrient for plant growth. At present, agriculture drives the nitrogen cycle [1] with current Nr concentrations higher than ever in the human era [2–3]. Ammonia oxidation, the first step of nitrification, is a biogeochemical process of global importance in natural and artificial ecosystems worldwide. In soils, ammonia-oxidizing bacteria (AOB) and ammonia-oxidizing archaea (AOA) co-exist, but their relative distribution and community composition may vary depending on the environmental conditions [4–7]. Soil properties and plant species influence the structure and function of microbial communities although the extent to which both factors contribute to microbial communities is not fully understood. Consequently, the microbial ecology of the nitrogen cycle in agricultural soils and the exploration of environmental factors that modulate the conversion of nitrogen between its different forms are issues of major interest.

AOA and AOB play a fundamental role in the interconnection between N fixation and N losses, catalyzing the oxidization of NH_4_^+^ to NO_2_^-^. These microorganisms show intrinsic and distinctive adaptations to natural habitats [8] and to environmental conditions such as NH_4_^+^ availability [9], temperature [10], and irradiance [11]. AOB and AOA have colonized similar environments worldwide but with different degrees of success in abundance, activity, and distribution patterns [12–15]. Both phyla encode for the enzyme ammonia mono-oxygenase (amo) that can be environmentally traced by the study of the gene coding for the alpha subunit of the enzyme (*amoA*). AOB and AOA respond differently to fertilization regimes [16], although AOB have been shown to be resposible of oxidizing most ammonium nitrogen applied in fertilizers [17]. As the composition of microbial communities may influence the rhizosphere functioning, understanding the factors driving the AOA and AOB communities, particularly in agricultural systems, may help us predict how these systems will respond to different fertilization rates and climate change.

In this study, we explored the nitrifiers assemblages in the cultivated olive (*Olea europaea* L. subsp. *europaea* var. *europaea*), one of the most ancient domestic cultivated plants [18] of great ecological and socioeconomic relevance in the Mediterranean area but vulnerable to global change impacts with expected changes in the regional and local distribution [19]. Olive groves dominate the landscape of Andalusia, the southernmost region of Spain, occupying 1.6 million-ha, i.e., 19% of the total surface area of the region [20–21]. This is the main olive growing area in the world, producing more than one-third of the world’s olive oil. Several studies have been focused on the influence of farm and soil management practices on soil erosion, physicochemical properties, water availability, nutrient losses, and crop yield in olive orchards [22–24]. However, until very recently only a few studies have addressed the effect of different agricultural practices, environment and soil physico-chemical properties on biological characteristics of olive orchard soils at a wide-region scale [25–27]. Understanding how these factors may influence microbial assemblages is essential because microorganisms have a central role in nutrient cycling and are very sensitivity to changes in environmental conditions or management practices. Consequently, microbes are good indicators for assessing changes in soil quality or soil recovery from disturbance or stress due to increasing fertilization rates and climate change scenarios.

In this work we studied for the first time the nitrifying assemblage (AOA, AOB, NOB) present in the rhizosphere of 96 olive orchards in Andalusia. We used quantitative PCR on AOA and AOB amoA gene, and fingerprinting and sequencing analyses of the 16S rRNA gene for AOA, AOB, and NOB. The dataset covered differences in farm (conventional vs. organic agriculture) and soil (cover crops vs. bare soil) management systems, irrigation regimen (rain-fed versus drip-irrigated), and climatic (altitude range from >1000 m altitude to the sea level) and edaphic characteristics (a range of one order of magnitude in carbon and nitrogen content, more than three orders of magnitude in pH (5.36–8.97), and different soil textures from sandy to clay soils). In addition, different olive varieties and ages were included. We aimed to identify and ranking the main factors driving composition and abundance within the nitrifying assemblage. We hypothesized a major effect by farm management systems (mineral fertilizers or organic manure) and a minor influence of soil textures and olive tree varieties. Interestingly, the overall results showed the opposite.

## Material and Methods

### Ethics Statement

No specific permits were required for the described field studies. Permission for sampling the olive orchards were granted by the landowner. The 96 olive orchards and wild olive havens sampled in this study have been included in previous studies [26–28] aimed to study bacterial, nematode and mycorrhizal communities of the olive rhizosphere. The sites are not protected in any way. The areas studied do not involve any species endangered or protected in Spain.

### Sites description and rhizosphere sampling

We sampled the rhizosphere of 90 commercial olive orchards from several southern areas in Spain (i.e., Jaén, Granada, Córdoba, and Sevilla, Table 1 and S1 Fig). The selected olive orchards were > 0.02 km^2^ area, under active olive production for at least 15 years, and submitted to a specific farm management systems for, at least, the last 5 years. The dataset covered differences in farm (conventional vs. organic agriculture) and soil (cover crops vs. bare soil) management systems, irrigation regimen (rain-fed versus drip-irrigated) and olive tree variety (mainly “Lechin”, “Manzanillo”, “Nevadillo”, and “Picual”). The orchards also differ in climatic (altitude range from >1000 m altitude in Jaen and Granada to the sea level) and physicochemical soil characteristics. Additionally to the 90 commercial olive orchards dataset, samples from six wild olive havens containing feral forms (i.e., secondary sexual derivatives of the cultivated clones or products of hybridization between cultivated trees and nearby oleasters) located in Córdoba and Cádiz were included (i.e. acebuches, *Olea europaea* subsp. *europaea* var. *sylvestris*). Rhizosphere soil samples were collected between May and July 2009 in the area of the canopy projection from four different points around each individual tree. Only young and active root samples were taken from each sampling point. Eight to ten trees per orchard were sampled, and all samples were thoroughly mixed to end with a single representative composite sample per orchard [26].

**Table 1:** Characteristics and code of olive orchard soils evaluated in this study.

Farm management system	Soil management system^a^	Irrigation system	Olive age	Variety	Province and soil orchard code
Acebuches (6)	CG (4)	Rain fed (6)	>30 (6)	Acebuches (6)	Córdoba: S19 (MACO), S31 (LOMCO), S32 (EPCO)
	CLT (2)				Cádiz: LO, LOBA, BAETICA
Organic (41)	LT (14)	Irrigated (24)	≤15 (12)	Gordal (1)	Córdoba: S1, S3-5, S8, S10, S17, S20-22, S24-25, S27-29, S61, S63-64, S66, S68, S70-71
	CG (6)	Rain fed (17)	15–30 (5)	Manzanillo (1)	Granada: S89
	CM (18)		>30 (24)	Nevadillo (7)	Jaén: S36-37, S40-41, S44-45, S73-74, S76, S78, S80, S82-83, S85, S87
	CLT (3)			Picual (27)	Sevilla: S47, S50-51
				Picudo (4)	
				Verdial (1)	
Conventional (49)	LT (30)	Irrigated (20)	≤15 (11)	Arbequina (4)	Córdoba: S2, S6-7, S9, S11, S18, S23, S26, S30, S59-60, S62, S65, S67, S69, S72, S91-93
	CG (1)	Rain fed (29)	15–30 (5)	Gordal (2)	Granada: S90
	CM (7)		>30 (33)	Hojiblanca (1)	Jaén: S12-16, S33-35, S38-39, S42-43, S46, S75, S77, S79, S81, S84, S86, S88
	CH (11)			Lechin (2)	Sevilla: S48, S49, S52-58
				Manzanillo (1)	
				Nevadillo (5)	
				Picual (24)	
				Picudo (2)	
				Royal (4)	
				Verdial (4)	

^a^Cover crop, grazing (CG); Cover crop, light tillage (CLT); Traditional light tillage (LT); Cover crop, mowing (CM); Cover crop, herbicide (CH)

Number of soils analyzed for each case are shown in parantheses

### Environmental analysis and DNA extraction

Rhizosphere soil was air-dried and sieved (2-mm to 5-mm mesh size) prior to soil physicochemical analyses that included soil texture, pH, organic carbon (SOC) and nitrogen (N) content, Extractable P, Exchangeable K (ppm), CO_3_Ca (%), Water content (%) and cation exchange capacity (CEC) determined as described by the official Agroalimentary Laboratory of Córdoba, Spain [26]. In addition to these physicochemical parameters, climatic factors were also considered (average temperature and rain annual regimes, and potential evapotranspiration), as well as altitude. The range of values explored for such parameters are shown in Table 2 and the complete dataset can be found in Montes-Borrego et al [26]. DNA was extracted from rhizosphere soil pellets (approximately 200 mg) prepared as previously reported [29], using the PowerSoil DNA Isolation Kit (MO BIO Laboratories, Inc., Carlsbad, USA).

**Table 2:** Summary of relationships between environmental factors (explored range of values are also shown; see also Table 1) and SCG (AOA), AOB and NOB microbial assemblages composition from the rhizosphere of olive orchards.

Factors^a^	Range	SCG (AOA)		AOB		NOB		AOB+NOB	
		*r* ^*2*^	*P*	*r* ^*2*^	*P*	*r* ^*2*^	*P*	*r* ^*2*^	*P*
*Physicochemical*									
CEC (meq/100 g)	2.84–43.47	0.1126	< 0.01	-	-	-	-	-	-
OM (%)	0.33–8.58	-	-	-	-	-	-	0.0781	< 0.05
Organic C (%)	0.19–4.98	-	-	-	-	-	-	0.0779	< 0.05
Organic N (%)	0.03–0.36	-	-	-	-	-	-	-	-
**C:N ratio**	6.26–16.08	-	-			-	-	**0.2425**	**< 0.001**
**pH (H**_**2**_ **O)**	5.36–8.97	**0.4121**	**< 0.001**	0.1223	< 0.05	0.1652	< 0.001	**0.2488**	**< 0.001**
**pH (KCl)**	4.54–7.95	**0.3649**	**< 0.001**	0.1119	< 0.05	0.1901	< 0.001	**0.3407**	**< 0.001**
**Clay (%)**	3.30–78.10	**0.3155**	**< 0.001**	-	-	0.1622	< 0.001	**0.3124**	**< 0.001**
**Sand (%)**	8.70–91.60	**0.2359**	**< 0.001**	-	-	0.1232	< 0.01	**0.2776**	**< 0.001**
Extractable P (ppm)	2.50–68.50	-	-	-	-	-	-	-	-
**Exchangeable K (ppm)**	38–950	**0.2062**	**< 0.001**	-	-			0.1335	< 0.01
**CO**_**3**_ **Ca (%)**	0–91.28	**0.2804**	**< 0.001**	0.1024	< 0.05	0.1477	< 0.01	**0.3164**	**< 0.001**
Water content (%)	0.28–15.63	0.068	< 0.05	-	-	0.131	< 0.01	0.107	**< 0.01**
**Soil texture**		**0.2766**	**< 0.001**	-	-	-	-	**0.2629**	**< 0.001**
*Climatic*									
Temperature _mean_ (°C)	12.8–18.4	-	-	0.0845	< 0.05	0.1152	< 0.01	0.1109	< 0.01
Temperature _max_ (°C)	30.0–38.1	0.1235	< 0.01	-	-	-	-	-	-
Temperature _min._ (°C)	-0.7–6.0	-	-	-	-	-	-	0.0765	< 0.05
**Total rain (mm)**	440–1100	0.0854	< 0.05	0.1388	< 0.01	**0.2013**	**< 0.001**	0.1409	< 0.001
Average rainfall (mm)	46.0–91.7	-	-	0.0722	< 0.05	0.133	< 0.001	-	-
ETP	712.5–985.9	-	-	-	-	0.1038	< 0.01	0.1103	< 0.01
Altitude (m)	13–1063	-	-	0.1011	< 0.05	-	-	-	-
*Agronomic*									
Orchard management system		-	-	-	-	-	-	-	-
Soil management system		-	-	-	-	0.0982	< 0.05	0.1184	< 0.01
Irrigation regimen		-	-	-	-	-	-	0.0458	< 0.05
Cover crop		-	-	0.0411	< 0.05	-	-	-	-
Plantation age		-	-	-	-	-	-	-	-
**Olive tree variety**		**0.2217**	**< 0.01**	-	-	-	-	**0.335**	**< 0.001**

^a^Cation exchange capacity (CEC); potential evapotranspiration (ETP)

Correlations with environmental factors (*r* ^*2*^) were obtained by fitting linear trends to the NMDS ordination for the different assemblages, and significance (*P*) was determined by permutation (nperm = 1000). Only relationships with the highest significant weight (r^2^>0.2 and P<0.05) are shown in bold. Empty spaces means not significant

### Archeal and Bacterial (AOB and NOB) 16S rRNA gene analysis by pyrosequencing

Bacterial and Archaeal analyses were carried out by massively parallel pyrosequencing of amplicons with the GS Junior 454 platform. The regions V1-V3 and V3-V5 were amplified with primers 8F-357R [30] and 344F-915R [31] for bacteria and archaea, respectively. Sequences were processed combining the Quantitative Insights Into Microbial Ecology (QIIME) [32] and the USEARCH toolkit [33–34]. After quality filtering and chimera checking, 289005 and 18591 reads were finally grouped into 15677 and 41 OTUs clustered at 97% identity threshold for bacteria and archaea, respectively. As raw counts can vary by orders of magnitude from the same sequencing run, data was normalized by rarefying the reads of all samples to the lowest number of reads in each library (1600 and 100 reads for bacteria and archaea, respectively). Unfortunately, most of the samples derived in a low number of archaeal reads at the final OTU table, and only 45 samples kept more than 100 sequences. Rarefaction at the same depth was repeat 100 times in order to address the loss of less abundant OTUs, and results were converged on an averaged OTU table. The 16S rRNA gene sequences were assigned to specific taxonomic groups after automatically alignment with SINA v1.2.11 [35], and were imported into the SILVA SSU Ref NR 115 database [36] in ARB [37] for further taxonomic refinement. Partial sequences were inserted in the original tree keeping the overall tree topology by using the Parsimony Interactive tool implemented in ARB. Sequence similarities were calculated using the ARB distance matrix tool [23]. AOB and NOB were analyzed by subtracting the identified OTUs from the averaged table.

### Archaeal 16S rRNA gene analysis by fingerprinting and cloning

The general structure of rhizosphere archaeal assemblages was investigated by fluorescent terminal restriction fragment length polymorphism (FT-RFLP) of PCR amplified 16S rRNA gene sequences using universal archaeal primers. PCR amplifications were carried out by a semi-nested-PCR using the specific primer pair ARCH21f/ ARCH958r and 10 ng of template for the first PCR reaction, and ARCH21f/ ARCH915r for second PCR reaction [38]. PCR products digested with *Hae*III and *Rsa*I were analyzed in a 3130XL genetic analyzer (Applied Biosystems, California, USA), and terminal restriction fragments (TRF) profiles were generated [29]. According to the shifts observed in TRF profiles, we selected eight samples to describe the phylogenetic composition by PCR cloning of the 16S rRNA gene using the universal archaeal primer set ARC344F/ ARC915R [39]. PCR products were purified (QIAquick Kit, Qiagen) and cloned with TOPO TA kit (Invitrogen) following manufacturer’s instructions. Sequencing was carried out using external facilities (http://www.macrogen.com) from the T7 priming site in the plasmid. A total of 189 refined sequences were processed with Mothur v.1.31.0 [40] to determine representative OTUs at 97% identity threshold. Phylogenetic affiliation was carried out as described above for pyrotag sequences.

### Factors shaping the structure of AOA, AOB and NOB communities

Non-metric multidimensional scaling analyses based on dissimilarities calculated using the Bray–Curtis index after Hellinger standardization were run with MetaMDS functions within the vegan package [41] using R version 3.1.2 (R Foundation for Statistical Computing, http://www.R-project.org/), and environmental vectors were fitted using the envfit routine. Correlations with agronomic, edaphic and climatic variables (*r* ^2^) were obtained by fitting linear trends to the NMDS ordination obtained for each microbial group and significance (*P*) was determined by permutation (nperm = 1000).

### amoA gene analysis of AOA and AOB by quantitative PCR

AOA and AOB abundances were estimated by quantitative PCR of amoA genes using primer pairs, experimental conditions and quantification analyses as reported before [7, 42]. Archaeal amoA gene was amplified with the primer sets amoA23F-amoA616R [10] and bacterial amoA gene with primers amoA1F-amoA2R [43]. Spearman's rank (r_s_) correlation analyses were run to investigate the relationship between the abundance of amoA gene copies per gram of rhizosphere soil and environmental parameters. The effect of the different agronomic factors on gene abundances was analyzed by one-way ANOVA of the four root-transformed data for both AOA and AOB. Then, Tukey’s HSD test was applied for pairwise comparison of means. Student’s t-test was carried out to compare the abundances between AOA and AOB. Shapiro-Wilk and and Levene statistics were used to test for normality and homogeneity of variances, respectively. All statistical analyses were run in the R environment (http://www.r-project.org/) using the data provided in S1 Table.

### Nucleotide Sequences Accession Numbers

AOB and NOB 16S rRNA gene sequences were submitted to the EMBL database under accession numbers HG940623- HG940662, and archaeal 16S rRNA gene sequences under accession numbers HG931399-HG931499. The entire dataset of reads were deposited in the Sequence Read Archive of Genbank under BioProject PRJNA276257, the metadata for each sample are available at NCBI in the BioSample database (accession numbers SAMN03371513- SAMN03371608), and the sequence data are deposited in NCBI’s Short Read Archive (SRA) under accession number SRP055539.

## Results

### Composition of nitrifying bacterial assemblages and environmental factors shaping AOB and NOB community structure (16S rRNA gene)

**Fig 1:**
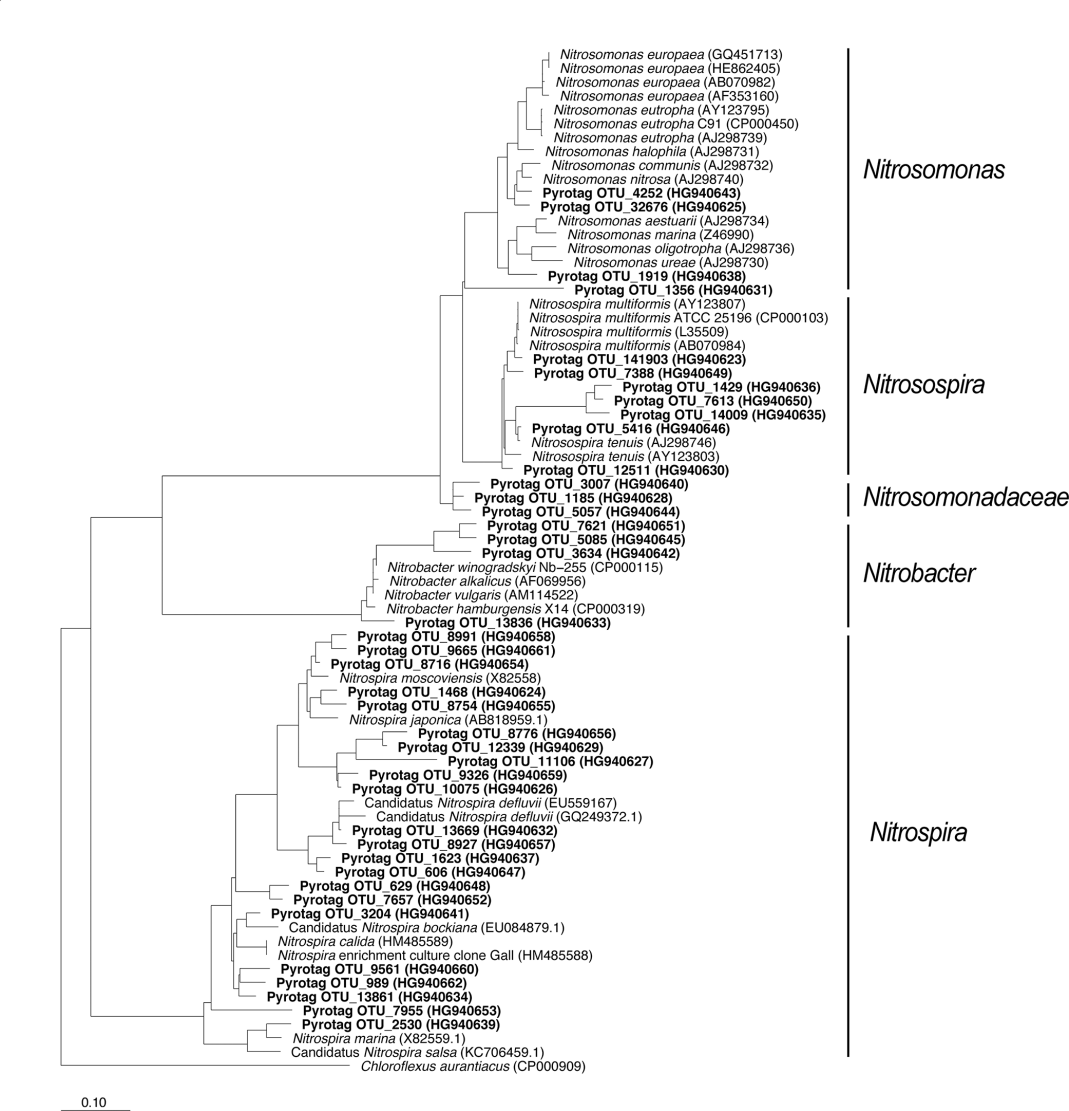
Maximun likelihood phylogenetic tree for the 16S rRNA gene of AOB (*Nitrosomonas*, *Nitrosospira* and *Nitrosomonadaceae*) and NOB (*Nitrobacter* and *Nitrospira*). Partial sequences (c. 350 bp, bold-faced) were inserted in the original tree by maximum parsimony keeping the overall tree topology. Bar scale: 10% divergence.

The AOB and NOB communities in the rhizosphere were dominated by *Nitrosospira* and *Nitrospira* species, respectively. AOB and NOB accounted for 0.4% and 0.2% of the total bacterial pyrotags sequences, respectively, and were detected in >95% of the rhizospheres sampled. Most (86%) of the sequences assigned to AOB were mainly within a single OTU with >99% identity with *Nitrosospira tenuis* (OTU_5416, Fig 1 and S2 Fig). The genus *Nitrosomonas* was very minor in abundance and only detected in c.10% of the olive rhizospheres. Conversely, sequences assigned to NOB were more equally distributed in two genera: 76% of the sequences were closely related to *Nitrospira* detected in 80% of the rhizospheres, and the remaining were closely related to *Nitrobacter* and detected in c. 55% of the rhizosphere samples (Fig 1 and S2 Fig).

**Fig 2:**
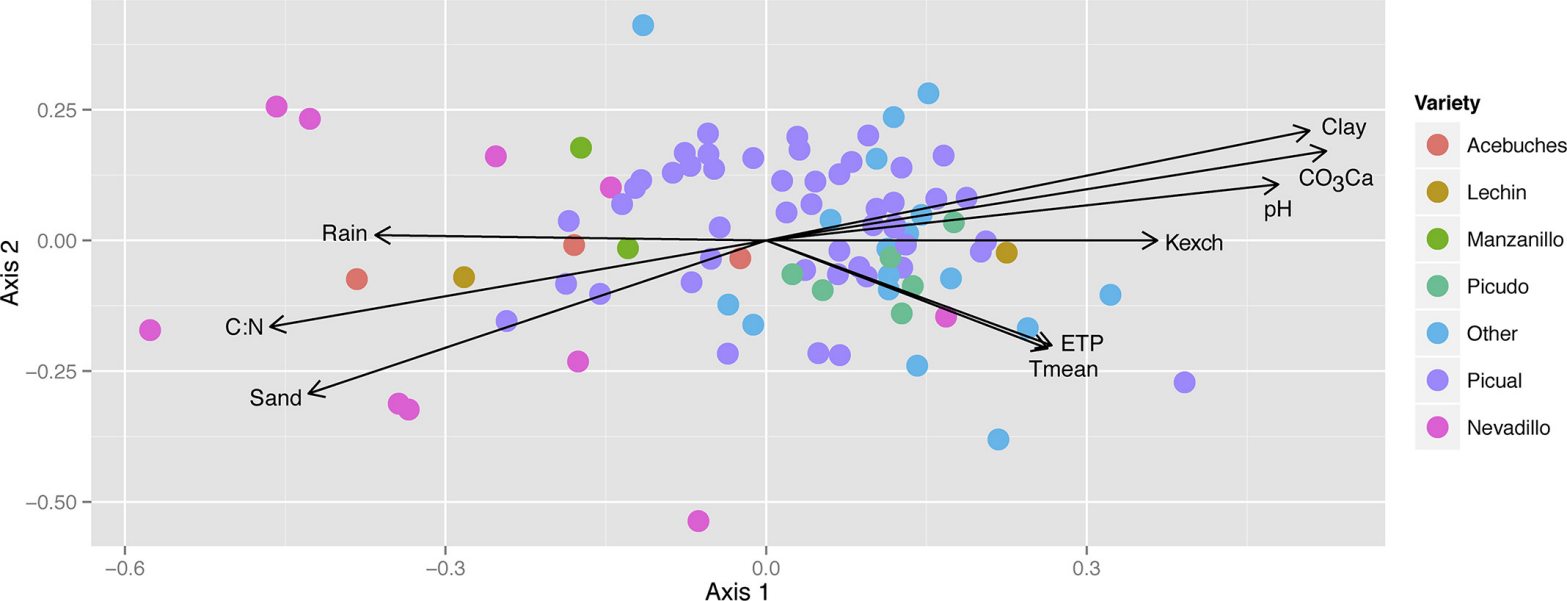
NMDS ordination analysis run for AOB and NOB assemblage composite composition, fitting on it the soil edaphic and climatic factors as environmental gradients significantly associated (*P*<0.05) with the ordination (see Table 2). Samples are highlighted according to the main olive varieties.

AOB and NOB community structure appeared strongly related to plant variety and different agronomic features (Fig 2). The NMDS ordination of AOB and NOB communities composition, separately, showed significant but low effects (*r* ^*2*^<0.20, *P*<0.001) for several edaphic factors (pH, clay, sand and water and calcium carbonate content) and climatic factors (mainly the total rain), and minor effect of some agronomic factors (Table 2). Conversely, the most significant values and higher effects were found when AOB and NOB rhizosphere communities were combined and analyzed together (*r* ^*2*^>0.20, *P*<0.001). With this combination, we found that different agronomic features acted as factors shaping the AOB and NOB assemblages, such as olive variety (*r* ^*2*^ = 0.335, *P*<0.001) (mainly olive varieties “Nevadillo” *vs*. “Picual”, see Fig 2) and soil management system (*r* ^*2*^ = 0.1184, *P*<0.01), as well as soil texture (*r* ^*2*^ = 0.2629, *P*<0.001).

### Composition of archaeal assemblages and soil factors shaping AOA community structure (16S rRNA gene)

**Fig 3:**
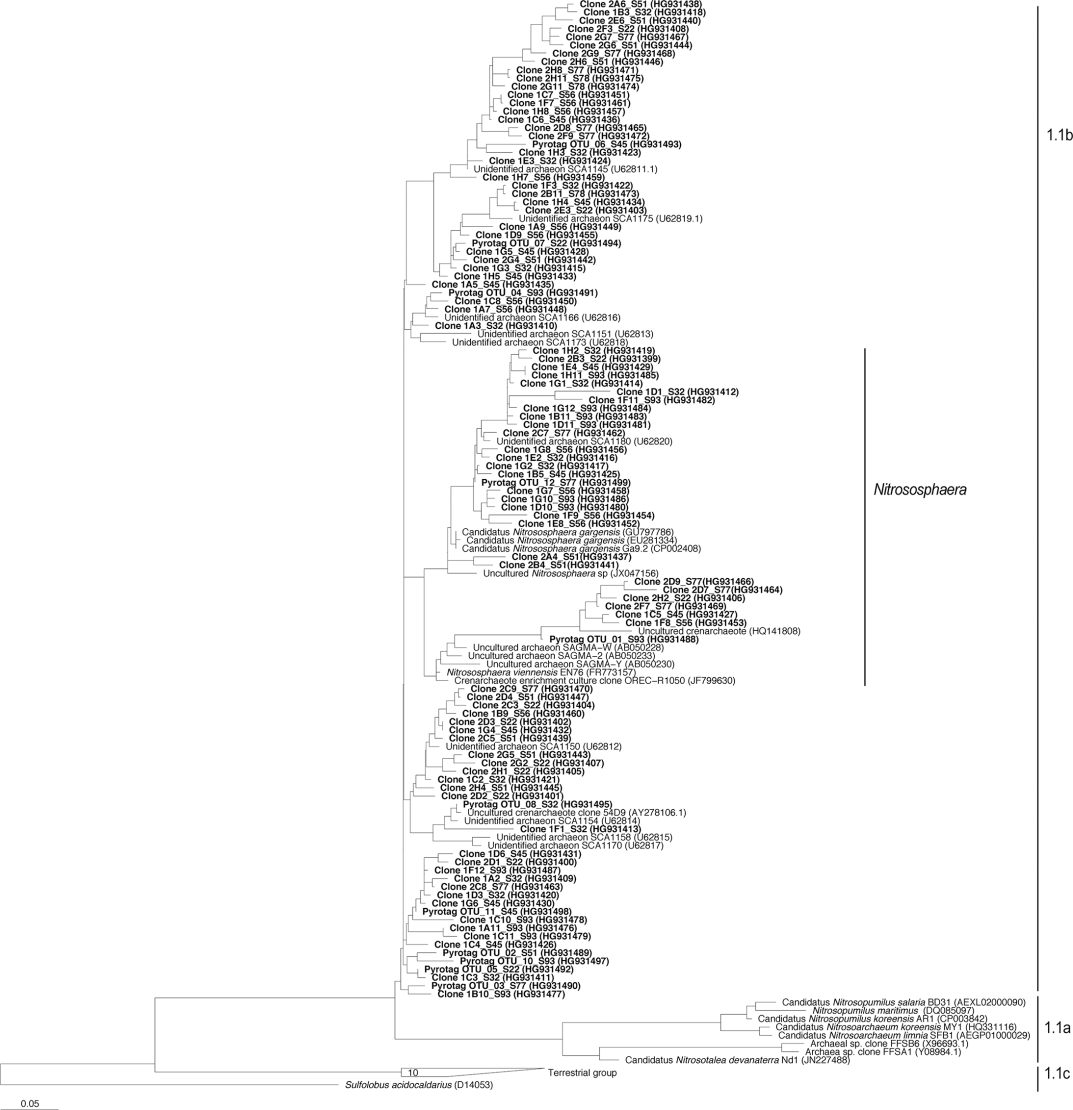
Maximun likelihood phylogenetic tree for the 16S rRNA gene of Thaumarchaeota 1.1b (soil crenarchaeotic group, SCG), including the *Nitrososphaera*-like clade. Partial sequences (c. 220 and 550 bp, bold-faced) were inserted in the original tree by maximum parsimony keeping the overall tree topology. Bar scale: 5% divergence

**Fig 4:**
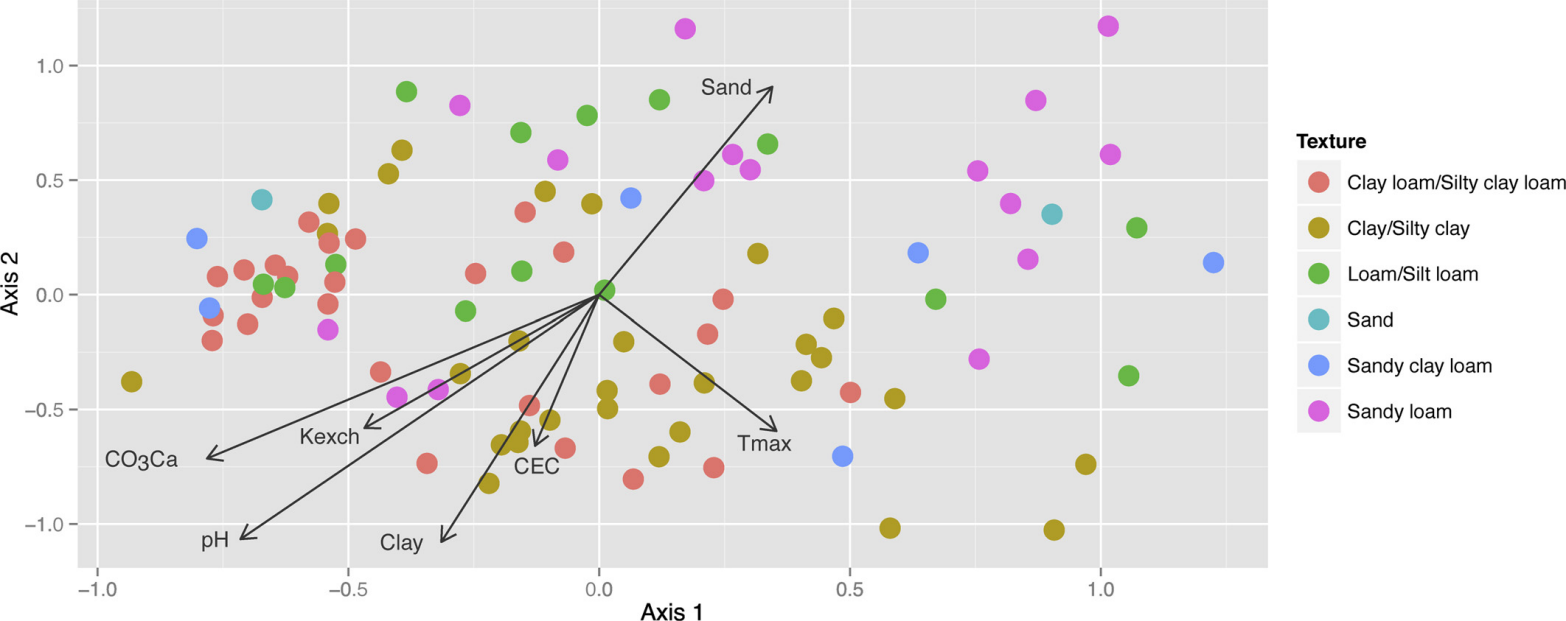
NMDS ordination analysis run for SCG (AOA) assemblage composition, fitting on it the soil edaphic and climatic factors as environmental gradients significantly associated (*P*<0.05) with the ordination (see Table 2). Samples are highlighted according to the soil texture properties.

Most of the archaea (>99% of the pyrotag sequences) were placed within the soil crenarchaeotic group, SCG (1.1b thaumarchaeota Fig 3 and S2 Fig) closely related to the AOA genus *Nitrososphaera*, and only a very few number of sequences matched *Euryarchaeota* of the groups *Halobacteria*, *Methanomicrobia*, and *Thermoplasmata*. The archaeal structure (mostly SCG) was further investigated by FT-RFLP of nested PCR-amplified 16S rRNA gene of large fragments (900 bp length) using HaeIII and RsaI restriction enzymes. Number of terminal restriction fragments (TRFs) ranged between 30 (*Rsa*I) and 43 (*Hae*III) depending of the olive orchard rhizosphere sample, and the hierarchical clustering analysis of the genetic fingerprints showed a distribution of soil samples in seven main clusters (S3 Fig). Representative soil samples within each cluster were selected for cloning and sequencing and the results were compared with those provided by pyrotag sequencing for the same dataset (S2 Table). The relative abundance of clone and pyrotag sequences for each sample showed little differences among groups with the exception of *Euryarchaeota* sequences that were not detected in the clone library, most probably because of the lower number of sequences analyzed. All the archaeal sequences obtained after cloning matched the soil crenarchaeotic group, SCG, and on average 25% of the sequences were most closely rooted to the genus *Nitrososphaera* (Fig 3). NMDS ordination analysis using the community composition derived from FT-RFLP data showed that the variables better explaining the AOA composition were pH (*r* ^*2*^>0.36, *P*<0.001), CO_3_Ca, clay and sand content and texture (*r* ^*2*^>0.27, *P*<0.001), followed by exchangeable K and olive tree variety (*r* ^*2*^ = 0.22, *P*<0.01), and minor effects were detected for CEC (*r* ^*2*^ = 0.1126, *P*<0.01), and yearly average maximum temperature (*r* ^*2*^ = 0.1235, *P*<0.01) (Fig 4 and Table 2). Similar NMDS distribution and association with soil factors were found with data derived from *Hae*III and *Rsa*I enzymes analyzed independently (*data not shown*).

### Agronomic, climatic and edaphic factors driving AOA and AOB abundances (qPCR of amoA genes)

The abundance of AOB amoA gene was negatively and significantly correlated with sand content (*r*_*p*_ = -0.27, *P*<0.05) and yearly average mean temperature (*r*_*p*_ = -0.35, *P*<0.001). Positive correlations were found with altitude (*r*_*p*_ = 0.44, *P*<0.001), pH (*r*_*p*_ = 0.25, *P*<0.05), and clay content (*r*_*p*_ = 0.32, *P*<0.01) (Table 3). Conversely, AOA abundance did not show any significant (*P*≥0.05) relationships with any of the former environmental variables but mean temperature (*r*_*p*_ = -0.24, *P*<0.05). Additionally, positive correlations (*P*<0.05) were found between AOA and organic nitrogen content and exchangeable K (Table 3).

**Table 3:** Spearman's rank correlation between the concentration of *amoA* genes copies and environmental parameters for AOA and AOB in the olive trees rizosphere.

Factors	AOA-*amoA*		AOB-*amoA*	
	r_s_	P	r_s_	P
*Physicochemical*				
CEC (meq/100 g)		ns		ns
OM (%)		ns		ns
Organic C (%)		ns		ns
**Organic N (%)**	**0.2720**	**< 0.05**		ns
C:N ratio		ns		ns
pH (H_2_O)		ns		ns
**pH (KCl)**		ns	**0.2552**	**< 0.05**
**Clay (%)**		ns	**0.3246**	**< 0.01**
**Sand (%)**		ns	**-0.2688**	**< 0.05**
Extractable P (ppm)		ns		ns
**Exchangeable K (ppm)**	**0.3102**	**< 0.05**		ns
CO_3_Ca (%)		ns		ns
Water content (%)		ns		ns
*Climatic*				
**Temperature**_**mean**_ **(**°**C)**	**-0.2416**	**< 0.05**	**-0.3536**	**< 0.001**
Temperature _max_ (°C)		ns		ns
Temperature _min._ (°C)		ns		ns
Total rain (mm)		ns		ns
Average rainfall (mm)		ns		ns
ETP		ns		ns
**Altitude (m)**		ns	**0.4356**	**< 0.001**

The effects of the different agronomic factors on the abundance of AOA and AOB in the olive rhizosphere were analyzed in more detail (Table 4). For AOA, significant differences in their abundance according to soil texture and olive tree variety were found, but no effect (*P*≥0.05) of orchard management system, irrigation and presence of a cover crop, or age of plantation was detected (Table 4). Tukey’s pairwise comparisons showed that clay loam soils significantly (*P*<0.05) hold the highest abundances of AOA than any other soil type. AOA were also significantly (*P*<0.05) more abundant in the rhizosphere of the olive variety “Picual” as compared to “Nevadillo”.

**Table 4:** Comparison of mean *amoA* genes concentrations for AOA and AOB assemblages in the olive trees rhizosphere.

Olive assemblage	Class	AOA-*amoA* ^*a*^	AOB-*amoA* ^*a*^
**Management**	Acebuches	3.16E+04	**3.32E+03** ^**a**^
	Conventional	2.66E+04	**5.33E+04** ^**b**^
	Organic	2.69E+04	**3.40E+04** ^**ab**^
**SMS**	LT	2.74E+04	**4.00E+04** ^**ab**^
	CLT	1.64E+04	**1.38E+04** ^**ab**^
	CG	1.37E+04	**6.35E+03** ^**a**^
	CH	2.99E+04	**7.98E+04** ^**b**^
	CM	4.23E+04	**5.90E+04** ^**b**^
**Texture**	Clay*	**1.36E+04** ^**b**^	4.53E+04
	Clay loam	**8.46E+04** ^**a**^	7.47E+04
	Loam	**9.76E+03** ^**b**^	2.81E+04
	Sand	**4.75E+04** ^**b**^	2.27E+04
	Sandy clay loam	**2.19E+04** ^**ab**^	2.24E+04
	Sandy loam	**6.19E+04** ^**ab**^	4.44E+04
	Silt loam	**1.36E+04** ^**ab**^	4.53E+04
Irrigation	Yes	2.83E+04	4.03E+04
	No	2.62E+04	3.39E+04
**Variety**	Acebuches	**3.16E+04** ^**ab**^	**3.32E+03** ^**b**^
	Arbequina	**4.60E+04** ^**ab**^	**7.89E+04** ^**abc**^
	Lechin	**4.04E+04** ^**ab**^	**1.19E+04** ^**abc**^
	Nevadillo	**6.19E+03** ^**a**^	**8.90E+03** ^**bc**^
	Picual	**4.42E+04** ^**b**^	**6.35E+04** ^**a**^
	Picudo*	**2.48E+04** ^**ab**^	**1.33E+05** ^**ac**^
	Royal	**2.79E+03** ^**ab**^	**1.36E+04** ^**abc**^
	Verdial	**1.12E+03** ^**ab**^	**2.03E+03** ^**abc**^
Cover	Yes	2.69E+04	3.38E+04
	No	2.74E+04	4.00E+04
Age (years)	≤15	3.16E+04	5.19E+04
	15–30	1.71E+04	2.58E+04
	>30	2.83E+04	4.03E+04

^a^Mean copies of amoA gene per gram of soil rhizosphere

Significant differences **within** each assemblage along the different agronomic factors were tested (p< 0.05, different letter a, b or c). Significant differences (p<0.05) in the mean concentrations **between** AOA and AOB assemblages were marked with an asterisk beside the corresponding class. For Soil Management Systems (SMS) code see Table 1.

For AOB, significant differences in abundances were found according to the orchard management systems and olive tree variety but no effect was observed for the remaining variables (Table 4). Significantly (*P*<0.05) lower AOB abundances were found in the rhizosphere of wild olives than in conventional cultivated olives trees, or with cover crop controlled by grazing as compared to mowing or herbicide. When AOA and AOB abundances were compared each other for the different categories within the different agronomic factors evaluated, significantly (*P*<0.05) lower abundances of the former were found only for clay soils and the olive variety “Picudo” (Student's t-test).

## Discussion

Ammonium concentrations in many soils have increased as a result of both changes in land-use with application of massive amounts of chemical fertilizers and increases in atmospheric reactive nitrogen concentrations [44], and this may influence the microbial ecology of the nitrification process [5, 15]. Ammonia oxidation is a critical step in the soil nitrogen cycle and can be differentially affected by the application of mineral fertilizers or organic manure. In olive cultivation N is applied annually at rates that reach 350 kg N/ha [45] usually during the winter in the forms of ammonium, urea or nitrate; with different forms of N being applied each year or during consecutive ones in the same orchard. However, annual application of N fertilizers to olive orchards have been shown unnecessary with the additional undesirable environmental effect that increases the leaching of nitrates to groundwater [45]. No matter which form of N is applied to olive soils that large amounts of it remains in the soil of the fertilized plots where NO_3_–N constitute the dominant mineral N fraction, probably because of the rapid hydrolysis of urea and the subsequent nitrification of NH_4_ [46]. The different chemical N forms that are introduced into olive agricultural soils can exert specific controls on the nitrifying microbial groups since ammonium availability is the most important factor determining the nitrification rate in soil, while nitrite and nitrate are known to inhibit nitrification [17]. Although this effect has major agronomic and environmental implications, information is lacking on the structure, diversity and population densities of AOB, AOA and NOB in olive agricultural soils and how these microorganisms may interact with plant N uptake and with the activities of other rhizosphere and endosphere microbial community members. This study represents the first exploration of the nitrifiers assemblages in the rhizosphere of cultivated olives in a wide region scale.

Although AOB have been reported to be stimulated under high ammonium conditions [47] we did not find significant differences between AOB and AOA densities along the different agronomical ranks explored. This could be related to the fact that instead of sampling the soil we focused our study in the rhizosphere. Plants together with N-assimilating microorganisms compete with nitrifiers for NH_4_-N, which may restrict or influence the growth of AOA and AOB in the rhizosphere [17]. Thus dynamic interactions of plant-ammonia oxidizers are expected in the rhizosphere [4, 48].

Interestingly, changes in NOB composition was better explained by environmental parameters than in the case of AOB. AOB were mostly composed by a single phylotype closely related to *Nitrosospira tenuis* along the different environmental ranges explored. Whereas the relative abundance of the NOB *Nitrobacter* was higher in sandy soils and with lower pH, the abundance of *N*. *tenuis* was modulated by the agronomic characteristics of the olive orchards sampled. When AOB and NOB were combined together many of the explanatory variables matched those that were significantly relevant to shape the AOA assemblage structure. These results suggest either a common response of NOB and AOA to environmental variables, or a higher versatility in AOB phylotypes than those of NOB and AOA. In any case, both groups of ammonia oxidizers have distinct physiological characteristics and ecological niches, with potential consequences in the nitrification process in the olive rhizosphere. The thaumarchaeota group 1.1b (SCG) has a global distribution being the dominant Archaea in a multitude of soils with just a few very abundant phylotypes [49–50]. In the rhizosphere soil we found a higher diversity of SCG than previously reported [50], probably belonging to the AOA group, which is in agreement with previous studies that have shown preferential distribution of soil ammonia-oxidizing archaea in the rhizosphere that in bulk soil [4, 51].

Even though our sampling effort was comprehensive, including orchards under different management systems, olive varieties, edaphic and climatic conditions, pooling the individual samples of each orchard in order to have one representative sample per orchard resulted in loss of the information on intra-site variability. Therefore, we were missing the intra-site variability at the local scale. However, major effects were detected for those parameters that showed variation at a larger spatial scale such as soil texture, pH, and olive variety.

Overall, olive variety and soil texture (in a continuous gradient from coarse textured to fine textured) affected not only the structure of AOA but also its abundance. Conversely, orchard management (conventional vs. organic), soil management and olive variety affected the abundance of AOB but not its structure. Interestingly, previous studies focused in the same olive orchards dataset had shown that, in general, the structure and diversity of arbuscular mycorrhizal fungi and plant parasitic nematodes in the olive rhizosphere did not differ between farming practices or soil management. In addition, olive variety, soil texture, sand and clay content, and soil pH were the main factors determining arbuscular mycorrhizal fungi and nematode assemblages [26, 28–29]. Thus, olive cultivar is emerging as one of the most important factors affecting the composition of rhizosphere-associated organisms, but abiotic (edaphic and climatic) factors seems to be important in maintaining the steady state of their populations [26, 28, 52]. In agreement with these results, in our study we observed that plant specific factors (i.e, olive genotype) and edaphic factors had more effect than farm management (organic vs conventional) practices. We observed however large variation across olive varieties and, although the olive varieties that we sampled were the most commonly grown in southern Spain (mainly “lechin”, “manzanillo”, “nevadillo”, and “picual”) we cannot ignore further effects by other varieties since each region or country grows specific varieties that makes each olive oil to have its own organoleptic characteristics. Interestingly, significant effects of the host variety and minor effect of soil type on both the AOB and AOA community structures has also been shown in potato fields [48]. Considering the fact that root exudates are highly plant species–specific, and the use of such specific compounds can shape plant-microbial communities interactions [53], one possible explanation that deserve more detailed investigations should consider the effects of root exudations (e.g. oxygen, carbon dioxide, dissolved organic matter) and differential plant-microbe interactions occurring at the variety level, as important factors shaping the composition and abundance of microbial nitrifiers in the olive rhizosphere. Whether or not these changes influence nitrogen utilization efficiency by olive crops remains to be determined.

## Supporting Information

S1 Fig Distribution of the 90 commercial olive orchards (*Olea europaea* var. *europaea*) and wild olive (‘Acebuches’, *Olea europaea* var. *sylvestris*) areas sampled in five provinces of southern Spain covering a wide range of physicochemical, climatic, and agronomic factors. (PDF)

S2 Fig Rank abundance plots for AOB, NOB, and SCG (AOA) 16S RNA gene OTUs present in the rhizosphere of *Olea europaea*. (PDF)

S3 Fig UPGMA cluster analysis of 16S rRNA gene T-RFLP fingerprintings obtained with restriction enzymes *Hae*III and *Rsa*I from the archaeal assemblages present in the rhizosphere of *Olea europaea* covering a wide range of physicochemical, climatic, and agronomic factors. (PDF)

S1 Table AmoA gene copies of AOA and AOB per gram of rhizosphere, and environmental parameters and agronomic factors for each soil. (PDF)

S2 Table Relative abundance (%) of the different Archaea groups found after 16S RNA gene cloning and 454 pyrotag analysis for eight selected soils. Taxonomic affiliation of OTUs was done based on the classification of automatic aligner SINA and phylogenetic analysis with ARB.
(PDF)
