# Supplementary figures and images for: Influence of Edaphic, Climatic, and Agronomic Factors on the Composition and Abundance of Nitrifying Microorganisms in the Rhizosphere of Commercial Olive Crops
Source: PLoS One. 2015 May 7;10(5):e0125787. doi: 10.1371/journal.pone.0125787 (PMC4423868; doi:10.1371/journal.pone.0125787)

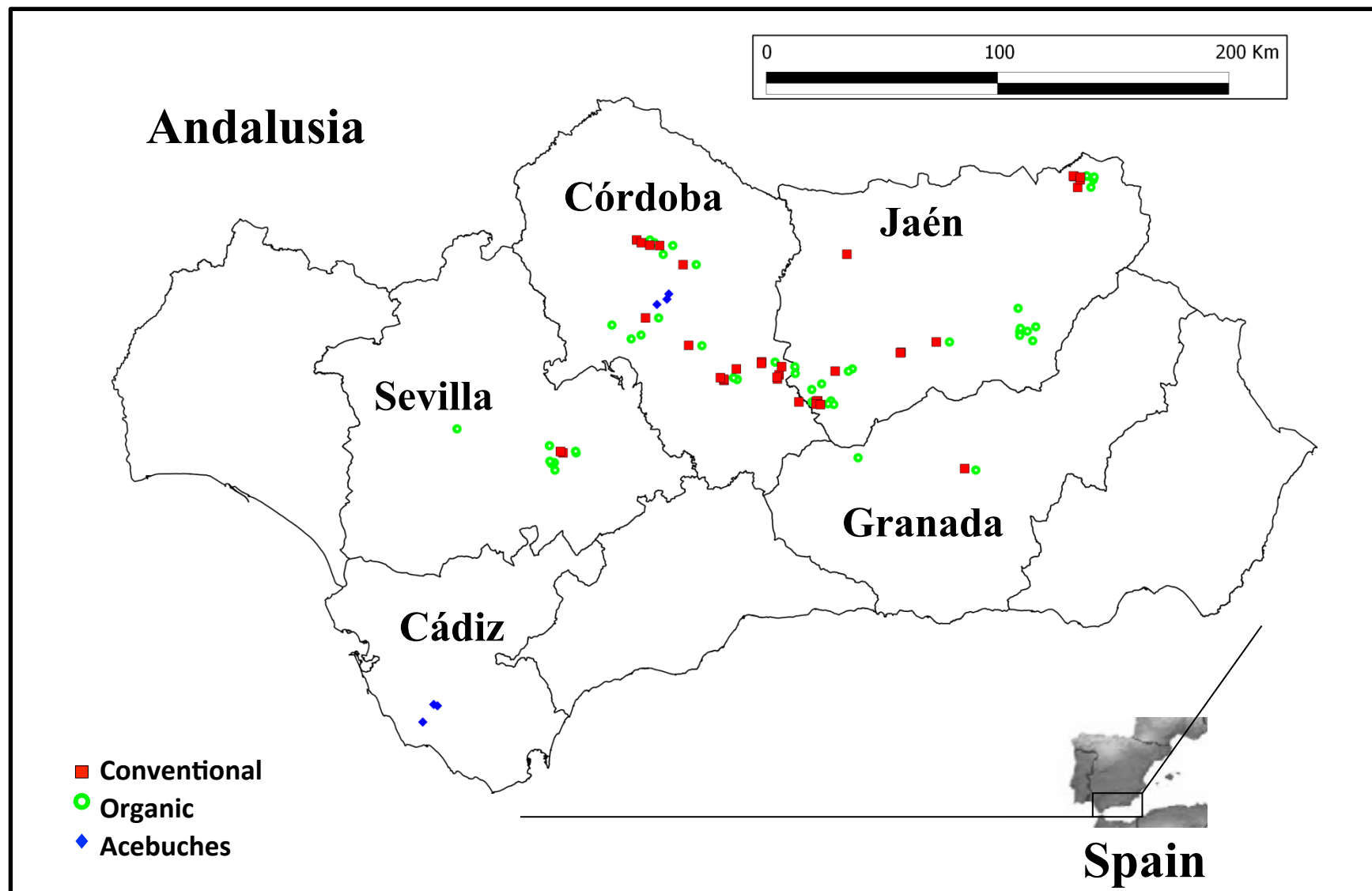

Supplement: S1 Fig — (PDF) [file pone.0125787.s001.pdf]

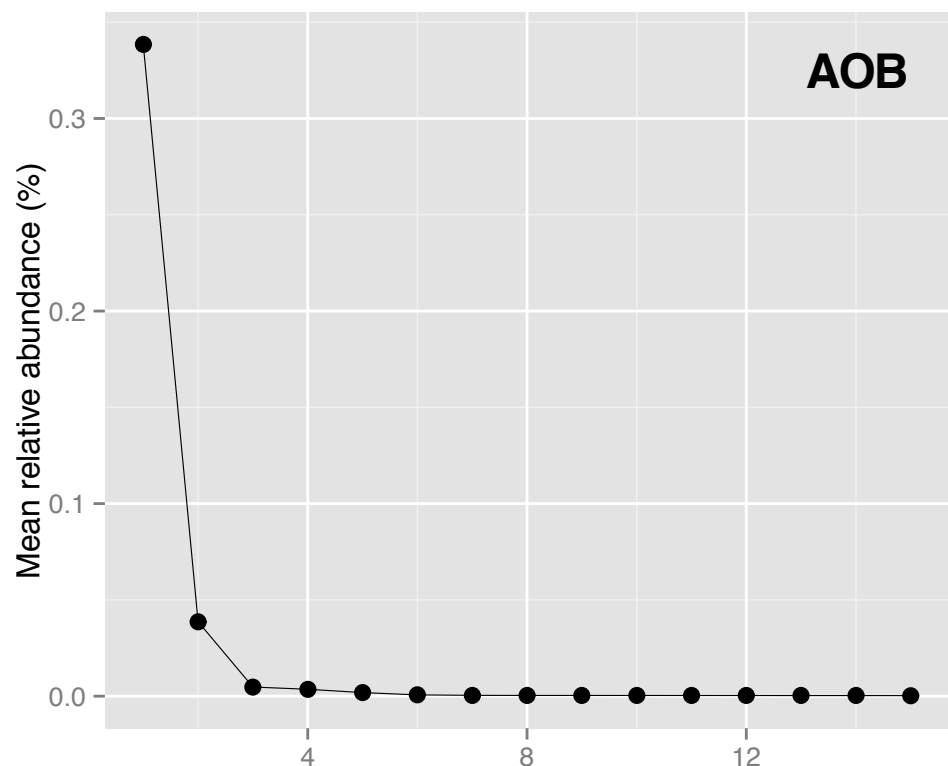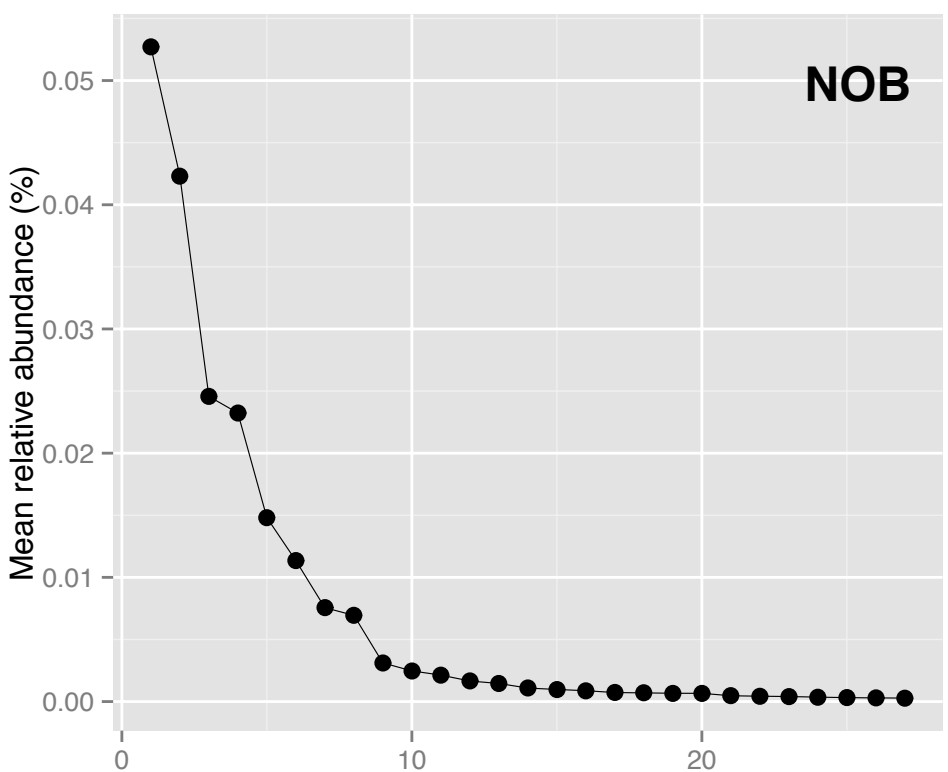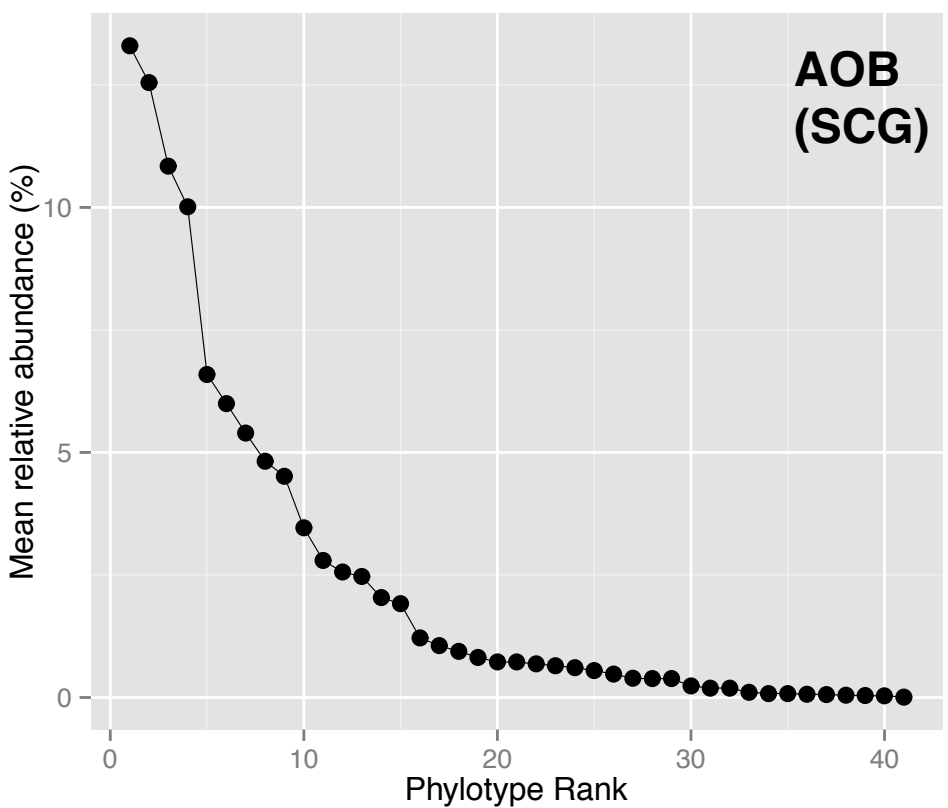

Supplement: S2 Fig — (PDF) [file pone.0125787.s002.pdf]
