# Supplementary material for: Influence of Edaphic, Climatic, and Agronomic Factors on the Composition and Abundance of Nitrifying Microorganisms in the Rhizosphere of Commercial Olive Crops
Source: PLoS One. 2015 May 7;10(5):e0125787. doi: 10.1371/journal.pone.0125787 (PMC4423868; doi:10.1371/journal.pone.0125787)
Supplement: S3 Fig — (PDF) [file pone.0125787.s003.pdf]

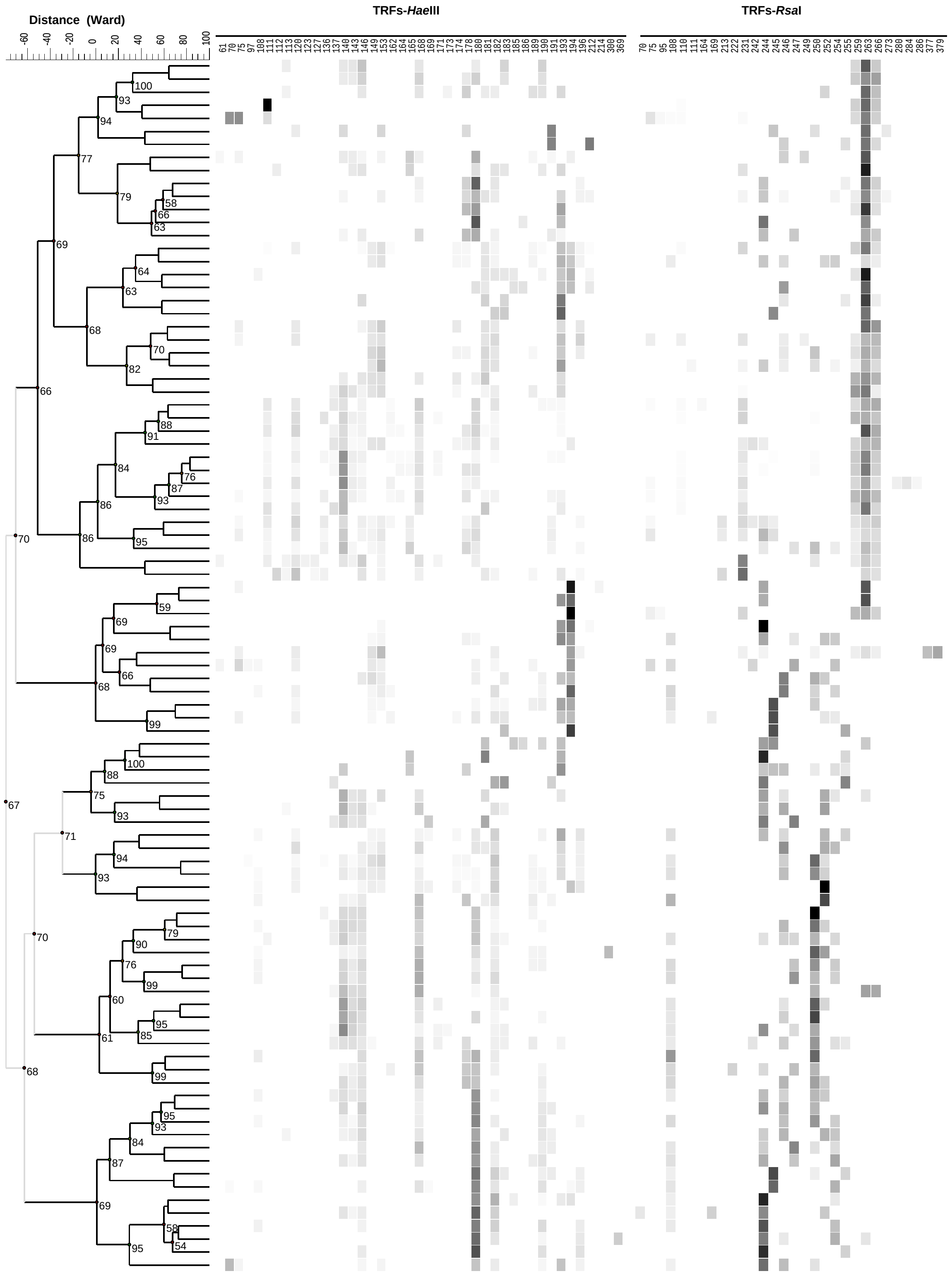

Orchard	Management	Texture	SMS	Irrigation	Cover	Age	Variety	Province
S32	A	Sandy loam	CLT	0	1	> 30 yr	Acebuches	Córdoba
S52	C	Clay	LT	0	0	> 30 yr	Gordal	Sevilla
S53	C	Clay	LT	0	0	> 30 yr	Gordal	Sevilla
S1	O	Clay	LT	0	0	< 15 yr	Picual	Córdoba
S2	C	Clay	LT	0	0	< 15 yr	Picual	Córdoba
S36	O	Sandy clay loam	CG	1	1	15-30 yr	Picual	Jaén
S80	O	Loam	CM	1	1	> 30 yr	Picual	Jaén
S68	O	Sandy loam	CG	0	1	> 30 yr	Nevadillo	Córdoba
S70	O	Loam	CLT	0	1	> 30 yr	Nevadillo	Córdoba
S86	C	Clay loam	LT	0	0	15-30 yr	Picual	Jaén
S87	O	Clay loam	CM	1	1	> 30 yr	Picual	Jaén
S38	C	Sandy loam	CH	0	1	> 30 yr	Picual	Jaén
S64	O	Sandy loam	CG	0	1	> 30 yr	Nevadillo	Córdoba
S85	O	Clay	CM	0	1	> 30 yr	Picual	Jaén
S13	C	Clay	CH	1	1	> 30 yr	Royal	Jaén
S77	C	Clay	LT	1	0	> 30 yr	Picual	Jaén
S56	C	Sandy clay loam	LT	0	0	15-30 yr	Manzanillo	Sevilla
S71	O	Sandy loam	LT	0	0	> 30 yr	Nevadillo	Córdoba
S31	A	Loam	CLT	0	1	> 30 yr	Acebuches	Córdoba
S54	C	Arena	LT	0	0	> 30 yr	Lechin	Sevilla
S6	C	Clay	LT	0	0	> 30 yr	Picudo	Córdoba
S7	C	Clay loam	CH	0	1	15-30 yr	Picual	Córdoba
S20	O	Clay	LT	1	0	< 15 yr	Picual	Córdoba
S22	O	Clay	CM	1	1	< 15 yr	Picual	Córdoba
S55	C	Clay	LT	0	0	> 30 yr	Verdial	Sevilla
S93	C	Clay loam	CH	1	1	< 15 yr	Arbequina	Córdoba
S12	C	Clay	CH	1	1	> 30 yr	Royal	Jaén
S9	C	Clay	LT	0	0	> 30 yr	Picudo	Córdoba
S3	O	Clay loam	CM	1	1	< 15 yr	Picudo	Córdoba
S8	O	Clay	CM	0	1	> 30 yr	Picudo	Córdoba
S10	O	Clay	LT	1	0	> 30 yr	Picual	Córdoba
S11	C	Clay	LT	0	0	> 30 yr	Picual	Córdoba
S5	O	Clay	CM	1	1	15-30 yr	Picual	Córdoba
S4	O	Clay loam	CM	1	1	> 30 yr	Picual	Córdoba
S14	C	Clay loam	LT	0	0	> 30 yr	Royal	Jaén
S23	C	Clay	LT	0	0	15-30 yr	Picual	Córdoba
S24	O	Clay	LT	1	0	15-30 yr	Picual	Córdoba
S21	O	Clay	CM	1	1	< 15 yr	Picudo	Córdoba
S47	O	Clay loam	CLT	0	1	> 30 yr	Gordal	Sevilla
S48	C	Clay loam	LT	0	0	> 30 yr	Verdial	Sevilla
S39	C	Sandy clay loam	CM	1	1	< 15 yr	Picual	Jaén
S63	O	Loam	CG	0	1	> 30 yr	Nevadillo	Córdoba
S16	C	Clay	CM	1	1	> 30 yr	Royal	Jaén
S79	C	Sandy loam	CM	0	1	< 15 yr	Picual	Jaén
S91	C	Sandy loam	CH	1	1	< 15 yr	Arbequina	Córdoba
S15	C	Clay	CM	1	1	> 30 yr	Picual	Jaén
S17	O	Loam	CM	1	1	< 15 yr	Manzanillo	Córdoba
S37	O	Sandy clay loam	LT	1	0	15-30 yr	Picual	Jaén
S65	C	Sandy loam	LT	0	0	> 30 yr	Nevadillo	Córdoba
S19	A	Sandy loam	CG	0	1	> 30 yr	Acebuches	Córdoba
S66	O	Sandy loam	LT	0	0	> 30 yr	Nevadillo	Córdoba
S61	O	Sandy loam	CG	0	1	> 30 yr	Nevadillo	Córdoba
S40	O	Sandy loam	CM	1	1	15-30 yr	Picual	Jaén
S72	C	Sandy loam	LT	0	0	> 30 yr	Nevadillo	Córdoba
S69	C	Loam	CG	0	1	> 30 yr	Nevadillo	Córdoba
S62	C	Sandy loam	CH	1	1	> 30 yr	Nevadillo	Córdoba
S45	O	Silt loam	LT	1	0	< 15 yr	Picual	Jaén
S46	C	Clay loam	LT	1	0	< 15 yr	Picual	Jaén
S30	C	Clay	LT	1	0	< 15 yr	Picual	Córdoba
S34	C	Clay	LT	0	0	> 30 yr	Picual	Jaén
S84	C	Clay loam	LT	1	0	> 30 yr	Picual	Jaén
S49	C	Loam	LT	1	0	< 15 yr	Verdial	Sevilla
S50	O	Clay loam	LT	1	0	< 15 yr	Picual	Sevilla
S33	C	Clay loam	LT	0	0	> 30 yr	Picual	Jaén
S73	O	Clay loam	CM	0	1	> 30 yr	Picual	Jaén
S25	O	Clay loam	CM	1	1	< 15 yr	Picual	Córdoba
S26	C	Clay loam	CH	1	1	> 30 yr	Hojiblanca	Córdoba
S28	O	Clay loam	LT	0	0	> 30 yr	Picual	Córdoba
S74	O	Clay loam	LT	0	0	> 30 yr	Picual	Jaén
S43	C	Clay loam	CH	0	1	> 30 yr	Picual	Jaén
S44	O	Clay loam	CM	1	1	> 30 yr	Picual	Jaén
S76	O	Clay loam	CM	1	1	> 30 yr	Picual	Jaén
S57	C	Sandy clay loam	LT	0	0	> 30 yr	Lechin	Sevilla
S89	O	Silt loam	CG	0	1	> 30 yr	Picual	Granada
S92	C	Clay loam	CH	1	1	< 15 yr	Arbequina	Córdoba
S90	C	Sandy loam	LT	1	0	> 30 yr	Picual	Granada
S41	O	Sandy clay loam	LT	1	0	> 30 yr	Picual	Jaén
S42	C	Clay loam	LT	0	0	> 30 yr	Picual	Jaén
S51	O	Loam	LT	1	0	< 15 yr	Verdial	Sevilla
S27	O	Loam	LT	0	0	> 30 yr	Picudo	Córdoba
S83	O	Clay loam	CM	0	1	> 30 yr	Picual	Jaén
S81	C	Clay loam	LT	0	0	> 30 yr	Picual	Jaén
S29	O	Clay	CM	1	1	< 15 yr	Picual	Córdoba
S59	C	Clay	CM	1	1	> 30 yr	Picual	Córdoba
S60	C	Clay loam	CM	1	1	> 30 yr	Picual	Córdoba
S67	C	Sandy loam	LT	0	0	> 30 yr	Nevadillo	Córdoba
S82	O	Clay	CM	1	1	> 30 yr	Picual	Jaén
S18	C	Loam	CH	1	1	< 15 yr	Arbequina	Córdoba
S58	C	Sand	LT	1	0	< 15 yr	Verdial	Sevilla
S75	C	Clay	LT	0	0	> 30 yr	Picual	Jaén
S88	C	Loam	LT	0	0	> 30 yr	Picual	Jaén
S78	O	Loam	CLT	1	1	< 15 yr	Picual	Jaén
S35	C	Clay	CM	0	1	15-30 yr	Picual	Jaén
