# Supplementary material for: Influence of Edaphic, Climatic, and Agronomic Factors on the Composition and Abundance of Nitrifying Microorganisms in the Rhizosphere of Commercial Olive Crops
Source: PLoS One. 2015 May 7;10(5):e0125787. doi: 10.1371/journal.pone.0125787 (PMC4423868; doi:10.1371/journal.pone.0125787)
Supplement: S1 Table — (PDF) [file pone.0125787.s004.pdf]

S1 Table. AmoA gene copies of AOA and AOB per gram of rhizosphere, and environmental parameters and agronomic factors for each soil

SampleID	Soil	amoA_AOB	amoA_AOA	Management	SMS	Texture	Irrigation	Variety	Cover	Age_Plantation	CEC	OM	C	N	C_N	pH	pH_ClK	Clay	Sand	Pexch	Kexch	CO3Ca	WaterConte	TotalRain	AverageRai	ETP	Temperatur	Temperatur	Temperatur	Altitude
S1	1	5600.98467	18815.9643	E	LT	Clay	No	Picual	No	15y	25.22	1.48	0.86	0.07	12.26	8.87	7.64	50.4	18	5.3	385	32.7979885	7.19030047	548.8667	66.0667	875.1	36.3	2.0333	17.1333	171
S4	4	11029.6567	23217.5394	E	CM	Clay loam	Yes	Picual	Yes	30y	28.26	2.28	1.32	0.15	8.82	8.75	7.58	35.6	23.1	22.9	435	53.0608941	4.52413218	542.6	60.6167	863.6333	36.15	1.0333	16.2833	402
S5	5	19441.3363	13224.5125	E	CM	Clay	Yes	Picual	Yes	15_30y	19.57	1.51	0.88	0.09	9.73	8.77	7.66	46.1	20.6	4.9	180	56.2978268	7.13138452	542.6	60.6167	863.6333	36.15	1.0333	16.2833	411
S9	9	207597.979	15298.5848	C	LT	Clay	No	Picudo	No	30y	24.13	1.68	0.97	0.11	8.86	8.52	7.57	44.5	30.8	4.9	270	41.1783477	5.09540784	543.3571	60.6571	865.8429	36.0857	1.3571	16.3571	391
S11	11	8344.82351	1747.90965	C	LT	Clay	No	Picual	No	30y	21.96	1.04	0.6	0.07	8.62	8.93	7.68	41.9	23.7	6.6	710	38.0301807	7.2108698	529.5	60.2333	872.2333	36.6667	0.4667	16.4	249
S12	12	24599.8965	1402.97434	C	CH	Clay	Yes	Royal	Yes	30y	23.48	1.38	0.8	0.1	8	8.52	7.59	45	20.6	27.5	680	41.7305006	4.95236101	609.1813	71.9938	907.8562	36.225	3.4937	17.5562	683
S13	13	40964.5214	905.682308	C	CH	Clay	Yes	Royal	Yes	30y	21.09	1.39	0.81	0.11	7.33	8.05	7.68	57.2	12.1	20.2	705	42.4858447	7.4399657	609.1813	71.9938	907.8562	36.225	3.4937	17.5562	737
S14	14	25744.9989	16985.9798	C	LT	Clay loam	No	Royal	No	30y	19.35	3.18	1.84	0.15	12.3	8.65	7.75	33.6	29.5	6.2	560	61.6123585	5.50458716	609.1813	71.9938	907.8562	36.225	3.4937	17.5562	893
S16	16	200	1341.61132	C	CM	Clay	Yes	Royal	Yes	30y	21.74	1.49	0.86	0.11	7.86	8.72	7.7	48	14.7	16	950	45.3018928	4.9475891	609.1813	71.9938	907.8562	36.225	3.4937	17.5562	628
S19	19	4215.80529	17178.1152	A	CG	Sandy loam	No	Acebuches	Yes	30y	19.56	4.39	2.55	0.21	12.13	7.39	6.35	14	56.5	10.6	280	0.49529729	2.52996005	630.4778	72.0722	895.7944	36.2056	3.3444	17.3167	269
S20	20	231577.512	147708.958	E	LT	Clay	Yes	Picual	No	15y	43.47	1.51	0.88	0.07	12.51	8.5	7.55	78.1	8.7	3.2	760	15.2301779	12.3797808	458.75	55.0667	909.15	35.9833	1.75	17.15	367
S21	21	51841.3448	21469.266	E	CM	Clay	Yes	Picudo	Yes	15y	18.91	1.1	0.64	0.08	7.98	8.95	7.71	40	30.9	3.4	420	50.0454799	4.68227425	458.75	55.0667	909.15	35.9833	1.75	17.15	372
S22	22	62384.9596	24059.6325	E	CM	Clay	Yes	Picual	Yes	15y	23.26	1.16	0.67	0.07	9.61	8.77	7.67	49.3	18.2	3.4	450	49.4382578	5.1421975	458.75	55.0667	909.15	35.9833	1.75	17.15	373
S23	23	10903.9624	5552.43967	C	LT	Clay	No	Picual	No	15_30y	27.61	0.94	0.55	0.06	9.09	8.64	7.53	56.6	13.4	12.5	570	42.1423585	7.69806479	458.75	55.0667	909.15	35.9833	1.75	17.15	371
S24	24	7842.26285	2828.01385	E	LT	Clay	Yes	Picual	No	15_30y	23.04	1.22	0.71	0.08	8.85	8.71	7.63	50.6	13.4	4	360	53.8172029	4.06065012	464.9143	55.7429	908.8143	35.9	1.7286	17.1571	354
S25	25	48489.5784	59605.9719	E	CM	Clay loam	Yes	Picual	Yes	15y	19.56	1.05	0.61	0.08	7.61	8.74	7.66	31.8	20.7	8.3	360	58.5190063	4.17919735	440.6667	51.8333	915.4333	35.6833	2.35	17.3	347
S26	26	66996.7528	29278.9526	C	CH	Clay loam	Yes	Hojiblanca	Yes	30y	16.08	1.57	0.91	0.11	8.28	8.86	7.91	35.5	25.8	5.8	280	67.3189605	5.15593826	440.6667	51.8333	915.4333	35.6833	2.35	17.3	379
S27	27	189860.766	43398.2429	E	LT	Loam	No	Picudo	No	30y	14.34	2.05	1.19	0.14	8.49	8.58	7.81	25.5	35.7	8.4	300	71.988432	2.52970094	471.1	54.8	907.79	35.58	2.08	17.1	357
S28	28	5061.12214	18547.0282	E	LT	Clay loam	No	Picual	No	30y	15.87	1.87	1.08	0.12	9.04	8.62	7.85	37.5	19.7	6.2	350	67.4396034	3.9912691	461.0333	54.6222	910.6667	35.6333	2.0111	17.1778	314
S29	29	168178.323	12178.6451	E	CM	Clay	Yes	Picual	Yes	15y	17.83	1.1	0.64	0.08	7.98	8.68	7.7	54.5	10.4	6.4	300	68.6345383	4.11942162	453.9125	53.125	913.0625	35.525	2.575	17.2625	403
S30	30	22685.1574	3747.53817	C	LT	Clay	Yes	Picual	No	15y	16.3	0.85	0.49	0.06	8.22	8.58	7.82	46.5	13	12.8	280	74.7658979	4.19223968	453.9125	53.125	913.0625	35.525	2.575	17.2625	408
S31	31	4817.25966	4077.1945	A	CLT	Loam	No	Acebuches	Yes	30y	15	2.51	1.46	0.11	13.24	8.4	7.41	16.1	51.2	2.8	285	2.22235892	1.96954315	666.0059	67.0118	877.4647	35.9824	2.9882	16.9412	362
S32	32	32432.2535	88277.6355	A	CLT	Sandy loam	No	Acebuches	Yes	30y	12.39	2.1	1.22	0.1	12.18	8.39	7.49	14	61.4	5.6	210	1.55689205	1.8398706	638.9333	71.1278	890.5667	36.1167	3.25	17.2056	274
S33	33	915858.874	54967.6462	C	LT	Clay loam	No	Picual	No	30y	22.61	2.26	1.31	0.13	10.08	8.39	7.62	31.9	25.8	3.6	360	50.7599596	4.09010692	701.4412	75.4294	712.5412	31.8882	1.1824	12.8294	1047
S34	34	60135.4012	19288.9685	C	LT	Clay	No	Picual	No	30y	26.52	0.91	0.53	0.06	8.8	8.49	7.6	52.1	12.1	10.6	530	39.1928279	6.57182291	509.3667	73.8	740.3167	34.0167	-0.7167	13.7	508
S36	36	17208.9137	11049.3844	E	CG	Sandy clay lo	Yes	Picual	Yes	15_30y	9.78	1.59	0.92	0.09	10.25	8.22	7.31	20.4	53.8	5.6	500	0.4595132	1.94659601	630.2667	70.9	836.7333	35.4333	1.5333	15.4	741
S37	37	91725.9229	108804.723	E	LT	Sandy clay lo	Yes	Picual	No	15_30y	10.87	1.4	0.81	0.08	10.15	8.41	7.41	24.6	52.2	4.3	480	1.71531647	1.98029857	630.2667	70.9	836.7333	35.4333	1.5333	15.4	738
S38	38	209114.489	23491.4748	C	CH	Sandy loam	No	Picual	Yes	30y	7.17	1.03	0.6	0.07	8.53	7.91	6.93	17.9	60	10.8	380	0	1.06908724	630.2667	70.9	836.7333	35.4333	1.5333	15.4	737
S39	39	36177.5105	1210.96473	C	CM	Sandy clay lo	Yes	Picual	Yes	15y	8.48	1.04	0.6	0.08	7.54	5.36	4.54	20.8	52.2	9.1	320	0.67612181	2.92457671	630.2667	70.9	836.7333	35.4333	1.5333	15.4	742
S41	41	66913.5902	31631.6419	E	LT	Sandy clay lo	Yes	Picual	No	30y	20.43	2.96	1.72	0.14	12.26	8.51	7.57	32.3	48.7	2.5	625	48.6201754	4.31714404	653.3375	70.5	836.6625	35.4	1.5	15.4125	812
S42	42	319519.31	169399.769	C	LT	Clay loam	No	Picual	No	30y	23.7	3.18	1.84	0.16	11.53	8.47	7.52	35.3	41.9	5.4	690	39.6116631	5.03672613	653.3375	70.5	836.6625	35.4	1.5	15.4125	808
S43	43	266581.887	328721.044	C	CH	Clay loam	No	Picual	Yes	30y	18.48	2.77	1.61	0.16	10.04	8.43	7.5	29.7	44.3	14.1	790	44.8313372	2.82485876	643.5833	69.9333	844.1	35.5167	1.6333	15.6	843
S44	44	107583.247	252901.674	E	CM	Clay loam	Yes	Picual	Yes	30y	20	2.5	1.45	0.15	9.67	8.6	7.62	38.1	39.3	5.6	870	34.0832224	4.68945394	649.12	71.86	843.32	35.62	1.6	15.52	838
S45	45	109618.845	124687.62	E	LT	Silt loam	Yes	Picual	No	15y	12.83	1.73	1	0.11	9.12	8.58	7.68	24.2	24.3	5	310	54.8257786	2.12636077	654.175	72.125	845.15	35.675	1.65	15.525	765
S46	46	34252.1653	220442.026	C	LT	Clay loam	Yes	Picual	No	15y	20	1.67	0.97	0.1	9.69	8.66	7.66	39.7	24.6	5.8	900	20.0861348	4.0725848	634.5	72.8	842.88	35.8	1.5	15.42	765
S49	49	8701.49996	3727.22057	C	LT	Loam	Yes	Verdial	No	15y	17.39	2.11	1.22	0.14	8.74	8.79	7.72	26.9	40.8	23.3	590	51.716449	3.31685208	527.1857	55.1857	939.4	36.3857	5.1286	18.1571	136
S50	50	24186.9611	27708.5017	E	LT	Clay loam	Yes	Picual	No	15y	17.83	2.38	1.38	0.14	9.86	8.62	7.82	27.7	43.4	5.2	345	33.9364386	6.15254952	531.4375	55.325	939.5125	36.4125	5.1625	18.1625	139
S53	53	671553.654	161430.547	C	LT	Clay	No	Gordal	No	30y	22.61	2.26	1.31	0.14	9.36	8.55	7.49	46.9	18.3	14.9	385	48.262293	5.89914367	532.1833	55.3167	918.5833	36.1833	4.9167	17.8667	155
S54	54	9008.21458	2936.96851	C	LT	Sand	No	Lechin	No	30y	2.84	0.33	0.19	0.03	6.38	7.08	6.32	3.3	91.6	4	45	0	0.27935748	537.2167	57.1167	941.425	36.3167	5.3	18.2333	150
S57	57	15507.1286	194110.618	C	LT	Sandy clay lo	No	Lechin	No	30y	15.87	1.34	0.78	0.09	8.64	8.54	7.49	21.6	61	13.3	390	30.5497406	3.41014777	542.3111	56.4889	929.1556	36.2556	5.0778	18.0222	151
S58	58	200	200	C	LT	Sand	Yes	Verdial	No	15y	5.87	0.74	0.43	0.05	8.58	6.92	5.95	7.1	91.4	52.4	290	30.0975646	0.76520338	537.1867	67.0733	948.6467	35.3533	4.7733	18.3733	148
S62	62	38593.0515	49383.9923	C	CH	Sandy loam	Yes	Nevaillo	Yes	30y	17.39	2.79	1.62	0.15	10.79	6.72	5.9	13.5	52.8	9.8	190	0.17315396	3.14355929	607.4	60.78	829.94	34.98	1.34	15.76	589
S63	63	9521.20273	7515.08142	E	CG	Loam	No	Nevaillo	Yes	30y	21.52	2.84	1.65	0.13	12.67	7.95	6.74	9.3	45	10.5	128	0	2.53216255	491.9692	77.3308	826.6385	33.2462	2.7846	15.6923	546
S64	64	3785.51703	1278.06777	E	CG	Sandy loam	No	Nevaillo	Yes	30y	20.65	3.45	2	0.15	13.34	7.02	5.77	8	60.8	8.2	129	0.19524355	3.15638451	584.2667	63.0333	829.7	35.0667	1.4833	15.7167	565
S65	65	8967.71026	20407.5963	C	LT	Sandy loam	No	Nevaillo	No	30y	14.35	3.35	1.94	0.16	12.14	5.89	5.12	6.4	58.5	6.2	210	0	3.30407847	584.2667	63.0333	829.7	35.0667	1.4833	15.7167	566
S66	66	200	5212.75472	E	LT	Sandy loam	No	Nevaillo	No	30y	12.61	2.92	1.69	0.12	14.11	7	5.79	6	75.5	13.8	114	0	1.54945166	599.8	62.54	829.52	35.06	1.34	15.72	623
S68	68	2842.17574	200	E	CG	Sandy loam	No	Nevaillo	Yes	30y	20.87	3.78	2.19	0.14	15.66	7.02	5.86	8.2	73.4	5.6	106	4.66911051	3.99087799	603.1167	62.0167	830.7833	34.9833	1.3667	15.75	481
S69	69	108794.684	16584.7283	C	CG	Loam	No	Nevaillo	Yes	30y	42.18	4.22	2.45	0.18	13.6	7.19	5.75	23.3	42.5	10	38	0	10.6368936	607.94	61.98	835.58	34.7	1.48	15.84	670
S70	70	13524.292	5842.66363	E	CLT	Loam	No	Nevaillo	Yes	30y	40	7.76	4.5	0.28	16.08	7.46	6.34	20.7	46.3	9.5	240	0	10.4734577	607.94	61.98	835.58	34.7	1.48	15.84	665
S71	71	19911.2054	1289.60811	E	LT	Sandy loam	No	Nevaillo	No	30y	11.08	3.79	2.2	0.15	14.66	7.93	7.07	6.4	67.9	6.4	190	1.85644031	4.29840876	617.6667	62.8667	870.7667	35.1	2.2667	16.6	393
S72	72	200	4566.29675	C	LT	Sandy loam	No	Nevaillo	No	30y	19.15	1.56	0.9	0.08	11.31	7.31	5.17	4.8	55.7	6.4	38	0.18171927	2.23903486	617.6667	62.8667	870.7667	35.1	2.2667	16.6	326
S73	73	76607.15	98816.2389	E	CM	Clay loam	No	Picual	Yes	30y	21.91	4.44	2.58	0.26	9.91	8.32	7.53	30.7	38.3	59.8	890	51.169018	7.58775685	491.9692	77.3308	826.6385	33.2462	2.7846	15.6923	1063
S75	75	46600.0336	14862.7406	C	LT	Clay	No	Picual	No	30y	22.26	2.46	1.43	0.14	10.19	8.18	7.59	44.6	23.7	4.7	420	48.159111	15.6289562	583.8923	73.5154	845.9154	32.9846	2.7538	16.0615	982
S76	76	128479.899	417077.056	E	CM	Clay loam	Yes	Picual	Yes	30y	26.26	2.3	1.33	0.16	8.34	8.69	7.45	38.4	37.3	4.5	375	24.2902844	7.4248927	583.8923	73.5154	845.9154	32.9846	2.7538	16.0615	708
S79	79	583349.231	298869.873	C	CM	Sandy loam	No	Picual	Yes	15y	8.43	1.63	0.95	0.1	9.45	6.13	5.29	13.7	59.6	14.6	230	1.10756665	2.60459393	604.8571	71.1	867.9	33.7286	3.9	16.3143	954
S81	81	23691.8419	59618.8854	C	LT	Clay loam	No	Picual	No	30y	13.22	2.29	1.33	0.16	8.3	8.69	7.77	30.2	35.7	7.5	465	28.4862082	6.16003387	548.42	48.56	829.58	33.68	2.78	15.7	638
S82	82	18390.6909	12802.4434	E	CM	Clay	Yes	Picual	Yes	30y	24.52	3.56	2.06	0.16	12.91	8.46	7.49	48.1	33.7	4.9	940	9.57237599	5.67053854	548.42	48.56	829.58	33.68	2.78	15.7	627
S84	84	87444.825	96052.5469	C	LT	Clay loam	Yes	Picual	No	30y	12.17	2.1	1.22	0.12	10.15	8.26	7.86	34.6	28.2	9.7	380	28.6927031	10.0482139	529.125	46.025	830.15	33.65	2.675	15.725	574
S85	85	150167.946	56807.1481	E	CM	Clay	No	Picual	Yes	30y	16.95	3.71	2.15	0.16	13.45	8.56	7.71	42.5	28.2	5	640	24.2882518	10.2262742	548.42	48.56	829.58	33.68	2.78	15.7	588
S86	86	61103.5884	33502.9388	C	LT	Clay loam	No	Picual	No	15_30y	15.65	1.52	0.88	0.09	9.8	8.68	7.62	38.4	30.5	4	410	24.6050114	8.01637755	548.42	48.56	829.58	33.68	2.78	15.7	581
S89	89	13756.2764	26430.4839	E	CG	Silt loam	No	Picual	Yes	30y	14.34	2.64	1.53	0.16	9.57	8.61	7.71	24	22.4	6.2	310	11.9696187	2.25464191	582.1167	71.85	769.8333	31.2667	2.5167	14.4167	967
S90	90	83718.0147	82863.5543	C	LT	Sandy loam	Yes	Picual	No	30y	12.6	1.82	1.06	0.09	11.73	8.74	7.74	19.6	56.2	10.7	325	11.3108916	2.29920294	582.1167	71.85	769.8333	31.2667	2.5167	14.4167	1018
S91	91	88248.3101	9690.65272	C	CH	Sandy loam	Yes	Arbequina	Yes	15y	7.17	0.54	0.31	0.05	6.26	8.46	7.4	12.4	61.4	30.2	185	0.17796798	1.26249874	590.6364	66.4909	910.2182	36.8818	3.3545	17.3091	134
S92	92	70285.6333	140655.759	C	CH	Clay loam	Yes	Arbequina	Yes	15y	18.47	1.51	0.88	0.1	8.76	8.65	7.71	34.8	36.9	23.3	625	21.0106321	4.21993556	571.7167	64.8167	917.2083	36.775	3.5	17.4583	81
BAETICA	BAETICA	200	157302.089	A	CG	Sandy clay lo	No	Acebuches	Yes	30y	32.61	8.58	4.98	0.36	13.82	7.81	7.08	21.7	68.6					1100	91.7		30	6	18	13
LO	LO	4561.25075	148552.913	A	CG	Sandy loam	No	Acebuches	Yes	30y	20.22	2.63	1.53	0.15	10.17	5.86	5.03	17.8	74.4					700	58.3		30	6	18	52
LOBA	LOBA	200	200	A	CG	Sand	No	Acebuches	Yes	30y	8.26	1.77	1.03	0.1	10.27	6.21	5.64	5.8	85					700	58.3		30	6	18	52
