# Supplementary material for: Influence of Edaphic, Climatic, and Agronomic Factors on the Composition and Abundance of Nitrifying Microorganisms in the Rhizosphere of Commercial Olive Crops
Source: PLoS One. 2015 May 7;10(5):e0125787. doi: 10.1371/journal.pone.0125787 (PMC4423868; doi:10.1371/journal.pone.0125787)
Supplement: S2 Table — Taxonomic affiliation of OTUs was done based on the classification of automatic aligner SINA and phylogenetic analysis with ARB. (PDF) [file pone.0125787.s005.pdf]

**S2 Table. Relative abundance (%) of the different Archaea groups found after 16S RNA gene cloning and 454 pyrotag analysis for eight selected soils.**

| | | S22 | S32 | S45 | S51 | S56 | S77 | S78 | S93 |
|---|---|---|---|---|---|---|---|---|---|
| **Number of sequences** | **Cloning** | 24 | 23 | 23 | 23 | 24 | 24 | 24 | 24 |
| | **Pyrotag** | 486 | 275 | 556 | 455 | 472 | 442 | 688 | 891 |
| **Soil crenarchaeotic group (1.1b)**[a] | **Cloning** | 87.5 | 69.6 | 78.3 | 78.3 | 70.8 | 79.2 | 100.0 | 41.7 |
| | **Pyrotag** | 85.2 | 80.7 | 88.3 | 79.3 | 87.3 | 84.4 | 94.5 | 56.2 |
| ***Nitrososphaera*** | **Cloning** | 12.5 | 30.4 | 21.7 | 21.7 | 33.3 | 20.8 | 0.0 | 58.3 |
| | **Pyrotag** | 10.7 | 19.3 | 11.7 | 20.2 | 8.9 | 15.6 | 5.5 | 42.9 |
| ***Euryarchaeota***[b] | **Cloning** | 0.0 | 0.0 | 0.0 | 0.0 | 0.0 | 0.0 | 0.0 | 0.0 |
| | **Pyrotag** | 4.1 | 0.0 | 0.0 | 0.4 | 3.8 | 0.0 | 0.0 | 0.9 |

[a]SCG without sequences most closely related to *Nitrososphaera* (see Fig. 3)
[b]*Halobacteria*, *Methanomicrobia*, *Thermoplasmata*

Taxonomic affiliation of OTUs was done based on the classification of automatic aligner SINA and phylogenetic analysis with ARB
